# A Low Creatinine to Body Weight Ratio Predicts the Incident Nonalcoholic Fatty Liver Disease in Nonelderly Chinese without Obesity and Dyslipidemia: A Retrospective Study

**DOI:** 10.1155/2020/4043871

**Published:** 2020-05-13

**Authors:** Jianxiong Lin, Jiehua Zheng, Xiaoqing Lin, Yexi Chen, Zhiyang Li

**Affiliations:** ^1^Department of Hematology and Oncology, Second Affiliated Hospital of Shantou University Medical College, Shantou, Guangdong 515041, China; ^2^Department of General Surgery, Second Affiliated Hospital of Shantou University Medical College, Shantou, Guangdong 515041, China; ^3^Department of Ultrasound, Second Affiliated Hospital of Shantou University Medical College, Shantou, Guangdong 515041, China

## Abstract

**Aim:**

A lower ratio of creatinine to body weight (Cr/BW) is considered the independent risk factor for incident nonalcoholic fatty liver disease (NAFLD). However, the relationship between the Cr/BW ratio and NAFLD among individuals without obesity and dyslipidemia and how this relationship is impacted by age are still ambiguous. Therefore, we explored the effect of the Cr/BW ratio on the incident NAFLD among Chinese without obesity and dyslipidemia of different age groups.

**Methods:**

A total of 9756 participants without NAFLD at baseline were included and grouped by the median value (1.32) of the Cr/BW ratio. Then, a further analysis was stratified by age (60 years old). The primary outcome was new-onset NAFLD.

**Results:**

After a median follow-up of 2.76 years, 844 (8.7%) participants developed NAFLD. The elderly had a higher person-years incidence rate and cumulative incidence rate than the nonelderly. A high Cr/BW ratio showed a lower cumulative incidence compared to a low Cr/BW ratio for the whole population (*P* = 0.039) and the nonelderly group (*P* = 0.008). After being adjusted for multivariate variables, the lower Cr/BW ratio was the independent risk factor for incident NAFLD in the nonelderly (HR 0.718, 95% CI 0.548-0.942), instead of the elderly.

**Conclusions:**

The Cr/BW ratio has a negative relationship with incident NAFLD among nonobese Chinese without dyslipidemia before the age of 60.

## 1. Introduction

With a global prevalence of 25% in the adult population, nonalcoholic fatty liver disease (NAFLD) has become the most common liver disease in the recent years [1]. NAFLD prevalence ranges from a low of 13% in Africa to a high of 32% in the Middle East [1]. In particular, the prevalence of NAFLD reaches up to 28% in Asia which was thought to be a nonepidemic area in the past [2]. NAFLD encompasses not only nonalcoholic fatty liver with hepatic steatosis as its distinguishing feature but also nonalcoholic steatohepatitis (NASH) characterized by the hepatic steatosis and hepatocyte damage [3]. Patients with NASH are considered to be at higher risk of progression to cirrhosis [4], although a significant number of patients with hepatic steatosis alone also progress to cirrhosis [5]. And the stage of hepatic fibrosis is the most important and significant predictor of both overall and liver-related mortality [6]. Furthermore, evidence is accumulating to suggest that NAFLD is associated with an increased risk of hypertension, diabetes mellitus (DM), and cardiovascular disease [7–9] and independent of traditional cardiovascular risk factors [10]. There is a global epidemic of obesity which is a well-known risk factor for the development of NAFLD [11–13]. But a previous review showed that 8-19% of patients with NAFLD are nonobese in Asia [14]. Beyond that, a significant number of nonobese NAFLD patients are presented with normal blood lipid levels [15]. Up to now, most studies about the relationship between NAFLD and risk factors have emphasized obesity and dyslipidemia; more attention should be paid to the incident NAFLD among the nonobese individuals with normal blood lipid levels for the purpose of preventing and managing NAFLD.

Increasing evidence reveals that the skeletal muscle index (SMI), defined as total appendicular skeletal muscle to body weight, is negatively related to the incident NAFLD [16–19]. And the muscle mass was measured by various convoluted methods, for instance, dual-energy X-ray absorptiometry [16, 17]. Under an ideal state, creatinine is excreted from the body at a relatively uniform rate which would be determined in large part by the total muscle mass [20–22]. Among individuals with normal renal function, the use of serum creatinine (Cr) has been proposed as an inexpensive and easily obtainable surrogate index to evaluate the skeletal muscle mass [21, 22]. Recently, an interesting novel index named the Cr to body weight (Cr/BW) ratio has been demonstrated to be quite useful for the prediction of T2DM and NAFLD [23, 24]. In fact, the positive relationship between Cr and body weight is even closer among nonobese individuals [20]. But it has not been previously reported whether the Cr/BW ratio could serve as a risk factor for incident NAFLD among nonobese individuals with normal blood lipid levels. Moreover, no data is available about the influence of age on the relationship between the Cr/BW ratio and incident NAFLD.

Therefore, we conducted a retrospective cohort study in China to explore the effect of the Cr/BW ratio on the incident NAFLD among nonobese individuals without dyslipidemia and how this relationship is impacted by age.

## 2. Materials and Methods

### 2.1. Study Design and Participants

The raw data was downloaded freely from the public database named “DATADRYAD” (http://Datadryad.org), which permits other researchers to reuse previously published data. By Dryad's terms of service, we cited the data package uploaded by Sun et al. in our present study [15, 25]. Sun et al. have clearly stated that the research protocol was approved by the ethics committee of Wenzhou People's Hospital [15]; therefore, there is no need for our study to get another ethical approval. And Sun et al. also have obtained verbal informed consent from all participants. A total number of 33153 initially NAFLD-free participants who attended an annual health check-up in Wenzhou People's Hospital during a period of 5 years (January 2010 to December 2014) were recruited. The following exclusion criteria were applied: (1) alcohol abuse (more than 140 g/week for men and 70 g/week for women); (2) a known history of chronic hepatic disease; (3) body mass index (BMI) ≥ 25 kg/m^2^ indicating obesity in China [14]; (4) a known history of taking antihypertensive, antidiabetic, or lipid-lowering agents; (5) being diagnosed as having dyslipidemia according to an elevated level of total cholesterol (TC) (>5.20 mmol/L), TG (>1.70 mmol/L), and LDL-C (>3.12 mmol/L) and declined levels of high-density lipoprotein cholesterol (HDL-C) (<1.03 mmol/L); (6) being diagnosed as having renal dysfunction (defined as Cr ≥ 133 mmol/L); (7) participants who were lost to follow-up; and (8) participants of incomplete data.

### 2.2. Data Collection

All participants were required to avoid exercise during the previous day and would undergo a medical examination in the morning after an overnight fast. A standardized questionnaire was used by a physician to obtain medical history and health habit. Standing height and weight were measured with wearing light clothing and no shoes. An automated sphygmomanometer was used to measure the blood pressure (systolic blood pressure (SBP) and diastolic blood pressure (DBP)) of all participants in a sitting position under a quiet environment. Biochemical variables, including alanine aminotransferase (ALT), aspartate aminotransferase (AST), gamma-glutamyl transpeptidase (GGT), alkaline phosphatase (ALP), total bilirubin (TB), albumin (ALB), globulin (GLB), Cr, blood urea nitrogen (BUN), fasting plasma glucose (FPG), uric acid (UA), TC, TG, HDL-C, and LDL-C, were measured with an autoanalyzer (Abbott AxSYM) with standard methods. More specific details can be obtained in the previous articles [15, 26].

### 2.3. Definitions and Outcomes

BMI (kg/m^2^) was calculated as the baseline body weight (kg)/height squared (m^2^), and the Cr/BW ratio was calculated as Cr (mmol/L) divided by body weight (kg). The outcome of interest was the incident NAFLD. NAFLD was diagnosed by abdominal ultrasonography in participants in reference to the suggestions of the Chinese Liver Disease Association [27]. In brief, the diagnostic criteria for NAFLD were diffuse hyperechogenicity of the close field echo in the liver (greater than the kidney or spleen) and an attenuated ultrasonic beam in the far field and any of the following items if they simultaneously occurred: ambiguous display of intrahepatic lacuna structure, mild to moderate hepatomegaly with blunt and round edges, decreased blood flow signal but normal blood distribution, and unclear or nonintact visualization of the capsule of the right lobe of the liver and diaphragm. Two experienced imaging specialists who were blinded to the examinee's history and the study design independently evaluated the ultrasonography images. Disagreements between the two imaging specialists were resolved by discussing with a third independent imaging specialist.

### 2.4. Statistical Analysis

All included participants were assigned into two groups by the median value (1.32) of the Cr/BW ratio: high Cr/BW ratio (>1.32) group and low Cr/BW ratio (≤1.32) group. At the same time, differences of baseline data between nonelderly (<60 years old) and elderly (≥60 years old) Chinese were evaluated. Continuous variables were expressed as mean ± standard deviation (SD) or median (interquartile range), and group comparison was conducted using one-way ANOVA or a nonparametric test as appropriate while categorical variables were expressed as number (percentage) and compared using the chi-squared test. The person-years incidence and cumulative incidence with 95% confidence interval (CI) of NAFLD were calculated, and the cumulative incidence stratified by the Cr/BW ratio was plotted as cumulative hazard curves using the Kaplan-Meier method and compared by the log-rank test. Multivariate Cox proportional hazards regression was used to evaluate the association of the Cr/BW ratio with the incident NAFLD by covering the variables with a *P* value less than 0.2 in the univariate analysis. A hazard ratio (HR) and 95% confidence interval (CI) would be reported. A subgroup analysis by age (nonelderly and elderly) was further conducted to reveal how the relationship between the Cr/BW ratio and NAFLD was impacted by age. Statistical significance was taken as*P* < 0.05for two-tailed analysis. All statistical analyses were performed by the use of SPSS V.25.0 software (SPSS Inc., Chicago, Illinois, USA).

## 3. Results

### 3.1. Baseline Characteristics of Participants

**Figure 1:**
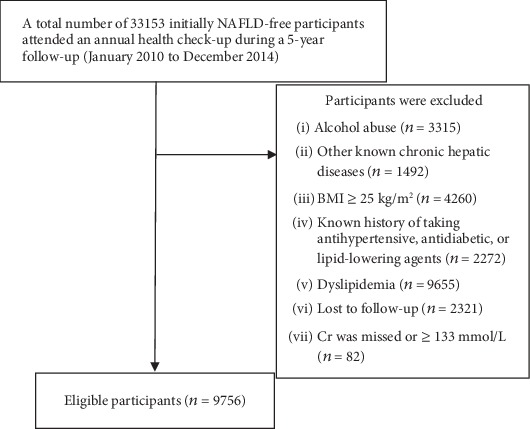
Flow chart. Abbreviations: NAFLD = nonalcoholic fatty liver disease; Cr = serum creatinine; *n* = number.

Overall, a total of 9756 participants who satisfied the inclusion and exclusion criteria were included in our study (Figure 1) and comprised 5011 (51.4%) male and 4745 (48.6%) female with a mean age of 42.38 years. The BMI was 21.02 ± 2.04 kg/m^2^, and the Cr/BW ratio ranged from 0.58 to 2.97. As compared with the high Cr/BW ratio group, the low Cr/BW ratio group had a significantly higher BMI, body weight, and TC. The age, proportion of male, SBP, DBP, ALP, GGT, AST, ALB, TB, BUN, Cr, UA, FPG, TG, and HDL-C of the low Cr/BW ratio group were statistically lower than those of the high Cr/BW ratio group. The difference of ALT, GLB, and LDL-C between the low Cr/BW ratio and high Cr/BW ratio groups did not reach statistical significance. Meanwhile, we assigned 1355 (14%) participants into the elderly group and 8401 (86%) participants into the nonelderly group, with a mean age of 70.44 years and 37.85 years, respectively. The proportion of male, BMI, SBP, DBP, ALP, AST, BUN, Cr, UA, FPG, and TG of the elderly group were statistically higher compared to those of the nonelderly group. On the contrary, the proportion of individuals with a low Cr/BW ratio and the value of ALB and GLB were lower in the elderly group than in the nonelderly group. Despite the fact that the same median was observed between different age groups, the difference of ALT was statistically significant. Two groups had the same body weight, GGT, TB, TC, HDL-C, and LDL-C. More specific baseline characteristics of different groups are presented in Tables 1 and 2.

### 3.2. Incidence Rate of NAFLD among Nonobese Individuals

**Figure 2:**
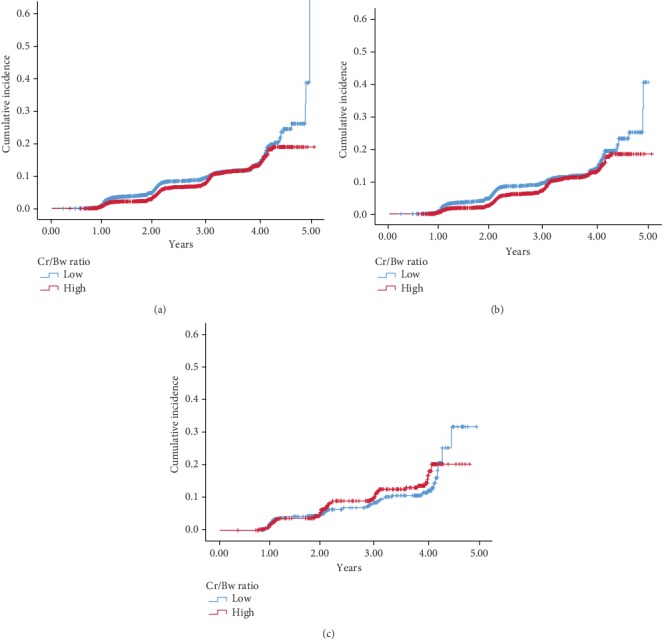
Cumulative incidence of nonalcoholic fatty liver disease for the whole population (a), nonelderly group (b), and elderly group (c).

Table 3 shows the incidence rate of NAFLD for the total study population and among various participant subgroups. During a mean follow-up of 2.76 years, 844 (8.7%) nonobese individuals were diagnosed with new-onset NAFLD. The total incidence rate of NAFLD was 313.45 per 10000 person-years, with a cumulative incidence of 8.65 (8.10-9.20). Moreover, the elderly group had a higher person-years incidence rate and cumulative incidence rate than the nonelderly group. For the whole population and the nonelderly group, a high Cr/BW ratio showed a lower cumulative incidence compared to a low Cr/BW ratio (Figure 2). The opposite result was observed in the elderly group; namely, the cumulative incidence was higher in the high Cr/BW ratio group than in the low Cr/BW ratio, but there was no significant difference in statistics (Figure 2).

### 3.3. The Relationship of the Cr/BW Ratio with the Incident NAFLD

The specific data is shown in Tables 4 and 5. According to the results of univariate analysis, there was a negative relationship between the Cr/BW ratio and incident NAFLD, with a HR of 0.866 (0.755-0.993). Multivariate Cox proportional hazards regression was conducted by covering the variables including Cr/BW ratio, age, gender, BMI, SBP, DBP, ALP, GGT, ALT, AST, ALB, GLB, TB, Cr, UA, FPG, TG, TC, TG, HDL-C, and LDL-C for their *P* value less than 0.2 in the univariate analysis. But the negative relationship between the Cr/BW ratio and incident NAFLD did not turn up in the multivariate analysis (HR 0.803, 95% CI 0.630-1.025). The effect of the Cr/BW ratio on incident NAFLD was further analyzed among different age groups. In model 1, a low Cr/BW ratio had a relationship with elevated risks of developing NAFLD for nonelderly participants (HR 0.817, 95% CI 0.703-0.948), but not elderly. After adjustment for gender and full adjustment, the low Cr/BW ratio was still a significant risk factor for incident NAFLD in model 2 (HR 0.812, 95% CI 0.699-0.942) and model 3 (HR 0.718, 95% CI 0.548-0.942). Another thing to mention was that Cr was not related to the incident NAFLD in different age groups (data not shown).

## 4. Discussion

For all we know, this retrospective cohort study is the first to demonstrate the relationship between the Cr/BW ratio and incident NAFLD among nonobese Chinese without dyslipidemia and how great is the influence of age on NAFLD in relation to the Cr/BW ratio. Our results indicated that there is a negative and independent influence of the Cr/BW ratio on the incident NAFLD among nonelderly participants. In addition, the incidence rate of NAFLD in the elderly group was statistically higher than that in the nonelderly group.

Although both obesity and dyslipidemia have been repeatedly discussed as well-known risk factors for developing NAFLD [12, 28], the prevalence of NAFLD ranges from 8% to 19% among Asian without obesity [14], and it was further confirmed that a significant number of nonobese NAFLD individuals are presented with normal blood lipid levels [15]. During a mean follow-up of 2.76 years, 844 (8.7%) nonobese individuals with normal blood lipid levels were diagnosed with new-onset NAFLD. It is quite important to identify another clinical index which is reproducible and inexpensive for predicting NAFLD among nonobese population without dyslipidemia. A recent retrospective study by Okamura et al. demonstrated that the Cr/BW ratio is negatively associated with incident NAFLD in both male and female [29]. Unfortunately, there remain significant uncertainties about the relationship of the Cr/BW ratio with incident NAFLD among nonobese individuals with normal blood lipid levels of different age groups. Therefore, we conducted a study including nonobese Chinese with normal blood lipid levels and revealed that the negative relationship of the Cr/BW ratio with incident NAFLD was only significant among the nonelderly.

Considering the fact that a positive correlation between Cr and the skeletal muscle mass has been established [20–22], it is desirable to replace the traditional measurement of muscle mass by Cr in clinical practice, especially in a developing country without a robust health care system. The decreased Cr/BW ratio, rather than Cr level, is associated with incident NAFLD in the recent study [24] and our study. The Cr/BW ratio has similar calculation formula to SMI which has been repeatedly discussed as a significant negative predictor of NAFLD [16–19]. When increased body weight was caused by decreased muscle mass and increased fat mass, particularly visceral fat accumulation could be a possible explanation for the difference between the Cr/BW ratio and simple Cr level. Therefore, the Cr/BW ratio is a more reliable indicator of skeletal muscle mass compared to the simple Cr level. Our research could enrich the recent evidence that the Cr/BW ratio serves as a predictive risk factor for NAFLD.

The mechanism about the relationship of the Cr/BW ratio with the incident NAFLD remains largely unknown. We suggest that this relationship could be mediated by insulin resistance because a lower Cr/BW ratio has been shown to increase the risk of DM in a recent study [23]. Concretely, SMI is a potent indicator of insulin resistance [30], and insulin resistance makes a great contribution to the incident NAFLD among nonobese individuals without DM [31]. As the largest insulin-sensitive organ, the skeletal muscle plays an important role in maintaining glucose homeostasis [32, 33]. Thus, sarcopenia, namely, a low SMI, is the contributing factor for insulin resistance both in nonobese and in obese individuals [34]. Moreover, a cross-sectional study found that sarcopenia is associated with increasing the risks of NAFLD independently of obesity and metabolic control [17], reminding us that other mechanisms are important in regard to the complex pathophysiological relationship between sarcopenia and NAFLD. Several myokines such as interleukin-6 and irisin which are secreted by the skeletal muscle regulate the metabolism of glucose and fatty acid [35, 36] and would lead to fat accumulation in the liver with the decrease in muscle mass [17]. Meanwhile, chronic inflammation is the common pathophysiological background of sarcopenia and NALFD. A variety of proinflammatory cytokines, like C-reactive protein, interleukin-6, and tumor necrosis factor-alpha, have a negative relationship with fat-adjusted skeletal muscle mass [37, 38], whereas disordered proinflammatory cytokine production is likely to play a role in the pathogenesis of NAFLD [39]. In addition, regular exercise has been proven to decrease the risk of incident NAFLD [17], and patients with sarcopenia tend to minimize activity, which means lower energy expenditure, followed by fat accumulation or even NAFLD [16]. At last, the serum vitamin D levels increase with advancing skeletal muscle mass and physical activity levels [40], but the serum vitamin D levels and its role in promoting NAFLD remain controversial [41, 42].

In our present study, elderly participants had a higher risk of developing NAFLD than nonelderly participants, but the Cr/BW ratio was irrelevant for incident NAFLD among the elderly. A previous study in Korea indicated a strong relationship of low muscle mass with NAFLD in middle-aged individuals [16]. Cr is excreted mainly through the kidney, primarily depending on the glomerular filtration rate which would decrease with advancing age, although the magnitude of the decline is highly variable [43]. At the same time, skeletal muscle mass decreases with advancing age. As shown in Table 2, Cr was higher in the elderly group than in the nonelderly group, but body weight of these two groups was the same. Therefore, the Cr/BW ratio may be inaccurate for the reflection of muscle mass and the prediction of NAFLD among the elderly.

Our study has some limitations. Firstly, there is an inevitable bias risk of retrospective study, which means that further prospective studies are needed to confirm our results. Secondly, insulin resistance is one of the most significant mechanisms for NAFLD among the nonobese individuals, but the initial study design did not allow for the measurement of the insulin levels. Lastly, some important variables like lifestyle, smoking, and waist circumference are unavailable, which may be strongly associated with NAFLD.

## 5. Conclusion

NAFLD is also a familiar metabolic disease among nonobese Chinese with normal lipid levels. The Cr/BW ratio is a negative and independent risk factor for the incident NAFLD among nonelderly participants without obesity and dyslipidemia. Prospective studies are required to evaluate the predictive value of the Cr/BW ratio for NAFLD.

## Figures and Tables

**Table 1:** Baseline characteristics of 9756 participants.

Characteristics	All participants	Cr/BW ratio		*P* value
		Low (≤1.32)	High (>1.32)	
Number (%)	9756	4854 (50)	4902 (50)	—
Age (years)	42.38 ± 14.66	41.76 ± 14.14	43.00 ± 15.13	<0.001
Male, *N* (%)	5011 (51.4)	2382 (49.1)	2629 (53.6)	<0.001
BMI (kg/m^2^)	21.02 ± 2.04	21.48 ± 1.90	20.57 ± 2.08	<0.001
Body weight (kg)	56.81 ± 8.30	57.82 ± 8.21	55.81 ± 8.26	<0.001
SBP (mmHg)	117.86 ± 15.88	116.93 ± 15.01	118.79 ± 16.64	<0.001
DBP (mmHg)	71.25 ± 9.94	70.96 ± 9.73	71.53 ± 10.14	0.004
ALP (U/L)	69.79 ± 21.78	67.27 ± 22.10	71.70 ± 21.33	<0.001
GGT (U/L)	19.00 (15.00-27.00)	18.00 (15.00-25.00)	20.00 (16.00-28.00)	<0.001
ALT (U/L)	15.00 (12.00-21.00)	15.00 (11.00-21.00)	15.00 (12.00-21.00)	0.170
AST (U/L)	22.19 ± 9.02	21.42 ± 8.37	22.78 ± 9.45	<0.001
ALB (g/L)	44.32 ± 2.71	44.24 ± 2.64	44.40 ± 2.78	0.006
GLB (g/L)	29.44 ± 3.85	29.48 ± 3.69	29.40 ± 4.00	0.300
TB (*μ*mol/L)	12.18 ± 4.92	11.68 ± 4.76	12.66 ± 5.02	<0.001
BUN (mmol/L)	4.43 ± 1.23	4.24 ± 1.14	4.61 ± 1.28	<0.001
Cr (mmol/L)	76.00 ± 17.16	65.60 ± 12.00	85.26 ± 15.21	<0.001
UA (*μ*mol/L)	262.84 ± 78.72	244.22 ± 71.10	281.28 ± 81.52	<0.001
FPG (mmol/L)	5.07 ± 0.70	5.03 ± 0.70	5.10 ± 0.69	<0.001
TC (mmol/L)	4.34 ± 0.53	4.35 ± 0.52	4.33 ± 0.54	0.012
TG (mmol/L)	0.97 ± 0.31	0.94 ± 0.31	0.99 ± 0.30	<0.001
HDL-C (mmol/L)	1.51 ± 0.30	1.50 ± 0.30	1.53 ± 0.30	<0.001
LDL-C (mmol/L)	2.12 ± 0.41	2.11 ± 0.40	2.12 ± 0.43	0.113
NAFLD (%)	844 (8.7)	478 (9.8)	366 (7.5)	<0.001

Note: continuous variables were presented as mean ± standard deviation or median (interquartile range), and categorical variables were presented as number (percentage). Abbreviations: Cr/BW = serum creatinine/body weight; BMI = body mass index; SBP = systolic blood pressure; DBP = diastolic blood pressure; ALP = alkaline phosphatase; GGT = gamma-glutamyl transpeptidase; ALT = alanine aminotransferase; AST = aspartate aminotransferase; ALB = albumin; GLB = globulin; TB = total bilirubin; BUN = blood urea nitrogen; Cr = serum creatinine; UA = uric acid; FPG = fasting plasma glucose; TC = total cholesterol; TG = triglyceride; HDL-C = high-density lipoprotein cholesterol; LDL-C = low-density lipoprotein cholesterol; NAFLD = nonalcoholic fatty liver disease.

**Table 2:** Baseline characteristics of 9756 participants stratified by age.

Characteristics	Nonelderly	Elderly	*P* value
Number (%)	8401 (86)	1355 (14)	—
Low Cr/BW ratio	4262 (50.7)	592 (43.7)	<0.001
Age (years)	37.85 ± 9.72	70.44 ± 6.81	<0.001
Male, *N* (%)	4026 (47.9)	985 (72.7)	<0.001
BMI (kg/m^2^)	21.00 ± 2.05	21.16 ± 2.00	0.008
Body weight (kg)	56.87 ± 8.37	56.43 ± 7.83	0.065
SBP (mmHg)	117.24 ± 15.40	121.75 ± 18.15	<0.001
DBP (mmHg)	71.10 ± 9.87	72.14 ± 10.30	0.004
ALP (U/L)	69.33 ± 21.34	72.58 ± 24.07	<0.001
GGT (U/L)	19.00 (15.00-27.00)	19.00 (15.00-27.00)	0.431
ALT (U/L)	15.00 (12.00-21.00)	15.00 (12.00-20.00)	0.034
AST (U/L)	20.05 ± 8.73	23.02 ± 10.60	0.002
ALB (g/L)	44.36 ± 2.70	44.10 ± 2.79	0.002
GLB (g/L)	29.38 ± 3.78	29.77 ± 4.23	0.001
TB (*μ*mol/L)	12.22 ± 4.98	11.97 ± 4.58	0.141
BUN (mmol/L)	4.38 ± 1.19	4.71 ± 1.36	<0.001
Cr (mmol/L)	75.64 ± 17.00	78.08 ± 18.07	<0.001
UA (*μ*mol/L)	261.69 ± 78.46	269.99 ± 79.96	<0.001
FPG (mmol/L)	5.04 ± 0.66	5.22 ± 0.85	<0.001
TC (mmol/L)	4.34 ± 0.53	4.36 ± 0.55	0.108
TG (mmol/L)	0.96 ± 0.31	0.99 ± 0.30	0.005
HDL-C (mmol/L)	1.51 ± 0.30	1.51 ± 0.30	0.788
LDL-C (mmol/L)	2.11 ± 0.41	2.12 ± 0.42	0.652
NAFLD (%)	719 (8.6)	125 (9.2)	0.405

Note: continuous variables were presented as mean ± standard deviation or median (interquartile range), and categorical variables were presented as number (percentage).

**Table 3:** Incidence rate of NAFLD of different groups.

Group	Number	Number of NAFLD	Cumulative incidence (95% CI)	Per 10000 person-years
Total	9756	844	8.65 (8.10-9.20)	313.45
Low Cr/BW ratio	4854	478	9.85 (9.26-10.43)	344.32
High Cr/BW ratio	4902	366	7.47 (6.65-7.98)	280.69
*P* value	—	—	0.039	—
Nonelderly	8401	719	8.56 (8.01-9.11)	310.09
Low Cr/BW ratio	4262	422	9.90 (9.32-10.49)	347.42
High Cr/BW ratio	4139	297	7.18 (6.67-7.68)	268.75
*P* value	—	—	0.008	—
Elderly	1355	125	9.23 (8.66-9.79)	337.92
Low Cr/BW ratio	592	56	9.46 (8.89-10.03)	325.07
High Cr/BW ratio	763	69	9.04 (8.48-9.61)	349.16
*P* value	—	—	0.311	—

Note: participants were assigned into the high Cr/BW ratio (>1.32) group and low Cr/BW (≤1.32) ratio group. The nonelderly participants were <60 years old, while elderly participants were ≥60 years old. Abbreviations: CI = confidence interval; Cr/BW = serum creatinine/body weight; NAFLD = nonalcoholic fatty liver disease.

**Table 4:** Univariate and multivariate Cox regression analysis for incident nonalcoholic fatty liver disease.

Characteristics	Univariate analysis		Multivariate analysis	
	HR (95% CI)	*P* value	HR (95% CI)	*P* value
High Cr/BW ratio	0.866 (0.755-0.993)	0.039	0.803 (0.630-1.025)	0.078
Age	1.007 (1.003-1.012)	0.002	1.008 (1.002-1.014)	0.007
Male	1.107 (0.967-1.267)	0.141	0.744 (0.625-0.885)	0.001
BMI	1.927 (1.842-2.016)	<0.001	1.571 (1.473-1.677)	<0.001
SBP	1.026 (1.022-1.029)	<0.001	0.988 (0.981-0.996)	0.002
DBP	1.048 (1.042-1.054)	<0.001	1.025 (1.013-1.036)	<0.001
ALP	1.012 (1.010-1.015)	<0.001	1.004 (1.001-1.008)	0.023
GGT	1.007 (1.006-1.008)	<0.001	1.001 (0.999-1.003)	0.176
ALT	1.007 (1.006-1.009)	<0.001	0.981 (0.962-1.000)	0.046
AST	1.012 (1.007-1.018)	<0.001	0.977 (0.968-0.985)	<0.001
ALB	0.972 (0.946-0.998)	0.035	1.024 (0.989-1.060)	0.184
GLB	1.021 (1.002-1.039)	0.029	1.008 (0.987-1.030)	0.435
TB	0.987 (0.972-1.003)	0.123	0.978 (0.960-0.996)	0.018
BUN	0.990 (0.938-1.046)	0.731	—	—
Cr	1.029 (1.026-1.033)	<0.001	1.003 (0.944-1.011)	0.544
UA	1.005 (1.004-1.006)	<0.001	1.000 (0.999-1.001)	0.788
FPG	1.322 (1.276-1.370)	<0.001	1.229 (1.137-1.329)	<0.001
TC	1.408 (1.229-1.612)	<0.001	0.632 (0.436-0.915)	0.015
TG	8.312 (6.736-10.257)	<0.001	2.341 (1.699-3.225)	<0.001
HDL-C	0.252 (0.195-10.326)	<0.001	0.641 (0.403-1.020)	0.060
LDL-C	3.309 (2.760-3.967)	<0.001	2.755 (1.741-4.358)	<0.001

Note: BUN was not included into multivariate analysis for the *P* value more than 0.2 in the univariate Cox analysis.

**Table 5:** Cox regression analysis for the association between the Cr/BW ratio and incident nonalcoholic fatty liver disease by age.

Group	Model 1		Model 2		Model 3	
	HR (95% CI)	*P* value	HR (95% CI)	*P* value	HR (95% CI)	*P* value
Nonelderly						
Low Cr/BW ratio	Ref		Ref		Ref	
High Cr/BW ratio	0.817 (0.703-0.948)	0.008	0.812 (0.699-0.942)	0.006	0.718 (0.548-0.942)	0.017
Elderly						
Low Cr/BW ratio	Ref		Ref		Ref	
High Cr/BW ratio	1.204 (0.840-1.724)	0.312	1.202 (0.839-1.722)	0.316	1.191 (0.635-2.236)	0.586

Note: model 1 is determined by univariate analysis; model 2 was adjusted for gender; model 3 was further adjusted for age, BMI, SBP, DBP, ALP, GGT, ALT, AST, ALB, GLB, TB, BUN, UA, FPG, TC, TG, HDL-C, and LDL-C. Abbreviations: HR = hazard ratio; CI = confidence interval.

## Data Availability

The data that support the findings of this study are openly available in the Dryad repository at 10.5061/dryad.1n6c4.
